# Association between serum NPTX2 and cognitive function in patients with vascular dementia

**DOI:** 10.1002/brb3.1779

**Published:** 2020-08-03

**Authors:** Keke Shao, Shiqin Shan, Wenwen Ru, Cuihua Ma

**Affiliations:** ^1^ Department of Neurology Shanxian Central Hospital Heze Shandong Province China

**Keywords:** biomarker, neuronal pentraxin 2, NPTX2, vascular dementia

## Abstract

**Objective:**

Neuronal Pentraxin 2 (NPTX2) has recently been widely reported as a novel biomarker for Alzheimer's disease (AD), but its correlation with vascular dementia (VaD) has not been elucidated. This study aimed to explore the correlation between NPTX2 and the cognitive function of VaD patients.

**Methods:**

112 VaD patients and 76 healthy controls were included in the study. Upon admission, clinical baseline data for all subjects were collected. Serum NPTX2 levels were determined using enzyme‐linked immunosorbent assay (ELISA). At the same time, the Montreal cognitive assessment (MoCA) scale was used to measure cognitive function. Multivariate regression analysis was used to determine the relationship between serum NPTX2 level and the cognitive function of VaD patients.

**Results:**

Compared with healthy controls, VaD patients had lower serum NPTX2 levels (*p* < .001). The results of Spearman's correlation analysis showed that serum NPTX2 levels in VaD patients were positively correlated with MoCA scores (*r* = .347, *p* = .042). The results of multivariate regression analysis showed that after adjusting for common risk factors, serum NPTX2 levels in VaD patients were still significantly associated with MoCA scores (*β* = 0.346, *p* = .039).

**Conclusions:**

Serum NPTX2 level was independently associated with cognitive function in patients with VaD. Serum NPTX2 level may be a novel predictor for cognitive function in VaD.

## 1 INTRODUCTION

Vascular dementia (VaD) is a syndrome of varying degrees of cognitive and memory impairment caused by cerebrovascular injury (Jia et al., 2018; Qian Wang, Yang, Zhang, Zhao, & Xu, 2020). VaD is a common form of dementia and affects millions of subjects around all the world. The World Health Organization (WHO) points out that there are currently about 35.6 million people with dementia worldwide, and the number is estimated to increase by 7.7 million annually (Xu et al., 2017). And among these dementia cases, VaD accounts for about 15%–20%, which is the second most common dementia subtype after Alzheimer's disease (AD). With the increase in human life expectancy, the number of VaD patients and the cost of treatment are expected to increase exponentially, which is attracting more and more attention (Llorens et al., 2020). Since currently VaD lacks specific treatments to slow down or prevent its progression, how to deal with the dementia caused by the aging population has become a public health problem that all human society must face.

Neuronal Pentraxin 2 (NPTX2), also named neuronal activity‐regulated pentraxin, is a secreted glycoprotein characterized by a cyclic multimeric structure (Osera et al., 2012; Tang et al., 2019). As a member of the pentraxins family, NPTX2 is highly conservative in evolution and is mainly expressed in the brain, spinal cord, and dorsal root ganglia (Moreno‐Rodriguez, Perez, Nadeem, Malek‐Ahmadi, & Mufson, 2020; Pribiag & Stellwagen, 2014). In vivo, NTPX2 exerts various neurological effects by combining with the transmembrane protein neuronal pentraxin receptor (NPTXR) (Chapman, Shanmugalingam, & Smith, 2019). NPTX2 was thought to play a vital role in transmitting neurotransmitters and maintaining synaptic plasticity (Kimoto, Zaki, Bazmi, & Lewis, 2015; Lee et al., 2017). Although the human NPTX2 gene was identified as early as 1995, little is known about its molecular spatial structure and biological function (Hsu & Perin, 1995).

In recent years, more and more studies on the correlation between NPTX2 and diverse neurological diseases have been reported. Till date, there are no data about the role of NPTX2 in VaD. Therefore, in our current study, we assume that NPTX2 is involved in the pathogenesis of VaD and its potential predicted value was investigated in VaD patients.

## 2 METHODS

### 2.1 Subjects

The cross‐sectional study was conducted in Shanxian Central Hospital between March 2017 and February 2020. Locals older than 60 years in Heze City were included in the study. A total of 188 subjects including 112 VaD patients and 76 controls were recruited in the study, and the demographic indicators (age, gender, and years of education) were recorded on admission. The diagnosis of VaD is made by an experienced neurologist. The diagnostic criteria of VaD refer to the National Institute for Neurological Disorders and Stroke (NINDS‐AIREN) and Diagnostic and Statistical Manual of Mental Disorders (DSM‐5) (Wang, Xu, Qi, Liu, & Zhao, 2020). Subjects with a clear history of cancer, brain trauma, acute cerebral infarction, mental disorders, alcohol and drug abuse, severe infection, organ dysfunction, or other types of dementia were excluded from this study. Each subject or their guardian signed an informed consent form. This study complies with the Declaration of Helsinki and was approved by the Clinical Research Ethics Committee of Shanxian Central Hospital.

### 2.2 Cognitive assessment

Montreal cognitive assessment (MoCA) was a widely used scale for global cognitive screens. The total score of MoCA is 30 points, which contains seven aspects such as orientation, executive function, language abilities, visuospatial abilities, short‐term and long‐term memory, abstraction, and attention. The lower the MoCA score, the worse the cognitive function. Generally, 26 points are used as the cutoff point for MoCA to diagnose cognitive impairment (Xu et al., 2019). The MoCA scores were assessed by trained attending physicians, who were unaware of the grouping and the clinical baseline data of all the subjects.

### 2.3 Laboratory assays

Venous blood was drawn early in the morning at least 8 hr after fasting. After centrifugation at 1200 g for 10 min at 4°C, the serum was separated immediately and frozen in a −80℃ refrigerator, and the biochemical indicators were subsequently determined. Blood biochemical index including free triiodothyronine 3 (FT3), free triiodothyronine 4 (FT4), thyroid‐stimulating hormone (TSH), fasting blood glucose (FBG), hemoglobin A1c (HbA1c), low‐density lipoprotein cholesterol (LDL‐C), triglycerides (TG), total cholesterol (TC), and high‐density lipoprotein cholesterol (HDL‐C) was measured by a blood automatic biochemical analyzer (VetScan HM5, Model No: 250735; M/s Abaxis, Pvt. Ltd). The serum concentrations of NPTX2 were determined using a commercial enzyme‐linked immunosorbent assay (ELISA) reagent (RD, Inc.). All experimental protocols refer to reagent instructions and previous research reports (Zhang, Tang, Hu, Wang, & Xu, 2020).

### 2.4 Statistical analysis

The clinical baseline data were analyzed with descriptive statistics. Data are expressed as n or mean ± SD. The comparison of clinical baseline data between groups was analyzed using Student's *t* test or chi‐square test. The Spearman correlation coefficient was used to correlate the MoCA score with clinical baseline data. A multivariate linear regression analysis was carried out for evaluation of NPTX2 with the MoCA scores. All statistical evaluations are statistically significant if the two‐tailed *p* value is less than .05. SPSS version 23.0 software (SPSS Inc, Chicago, IL, USA) was used for all the analyses in our current study.

## 3 RESULTS

### 3.1 Clinical baseline data

The study included 112 VaD patients and 76 healthy controls at the Shanxian Central Hospital from March 2017 to February 2020. Clinical baseline data including demographic indicators and blood biochemical indicators of all subjects were recorded after admission, and they are summarized in Table 1. The differences in demographic indicators including age (73.2 ± 5.3 vs. 72.9 ± 6.0), gender (male/female: 64/48 vs 45/31), and education years (8.0 ± 2.4 vs 8.1 ± 2.6) between the groups are not significant (*p* > .05). There was also no significant difference between the two groups in the comparison of blood biochemical indexes including FT3, FT4, TSH, FBG, HbA1c, LDL‐C, TC, TG, and HDL‐C. However, compared with healthy controls, VaD patients had significantly lower serum NPTX2 levels (196.8 ± 16.5 vs. 242.6 ± 19.4, pg/ml) and MoCA scores (22.8 ± 2.4 vs. 27.8 ± 1.3), and the difference between them was statistically significant (*p* < .05).

**TABLE 1:** Baseline characteristics of all subjects

	VaD (*n* = 112)	Control (*n* = 76)	*p*
Age, years	73.2 ± 5.3	72.9 ± 6.0	.719
Gender, male/female	64/48	45/31	.778
Education, years	8.0 ± 2.4	8.1 ± 2.6	.787
FT3, pmol/L	4.28 ± 0.34	4.33 ± 0.38	.347
FT4, pmol/L	12.65 ± 1.19	12.57 ± 1.23	.656
TSH, mIU/L	0.45 ± 0.08	0.43 ± 0.09	.112
FBG, mmol/L	5.26 ± 0.51	5.28 ± 0.60	.806
HbA1c, mmol/L	5.44 ± 0.62	5.39 ± 0.67	.600
LDL‐C, mmol/L	2.51 ± 0.22	2.48 ± 0.25	.387
TG, mmol/L	1.62 ± 0.17	1.60 ± 0.18	.441
TC, mmol/L	4.85 ± 0.67	4.83 ± 0.71	.845
HDL‐C, mmol/L	1.33 ± 0.12	1.35 ± 0.11	.248
NPTX2, pg/ml	196.8 ± 16.5	242.6 ± 19.4	<.001
MoCA	22.8 ± 2.4	27.8 ± 1.3	<.001

Abbreviations: FBG, fasting blood glucose; FT3, free triiodothyronine 3; FT4, free triiodothyronine 4; HbA1c, hemoglobin A1c; HDL‐C, high‐density lipoprotein cholesterol; LDL‐C, low‐density lipoprotein cholesterol; MoCA, Montreal cognitive assessment; NPTX2, neuronal pentraxin 2; TC, total cholesterol; TG, triglycerides; TSH, thyroid‐stimulating hormone; VaD, vascular dementia.

### 3.2 Spearman's correlation analysis

The correlation analysis results of the MoCA score of VaD patients and clinical baseline data are presented in Table 2. The results of Spearman's correlation analysis showed that the serum NPTX2 level of VaD patients was significantly positively correlated with the MoCA score (*r* = 0.347, *p* = .042). However, other clinical baseline data of VaD patients were not significantly correlated with MoCA scores (*p* > .05).

**TABLE 2:** Correlation coefficients between MoCA and baseline characteristics in patients with VaD

	*r*	*p*
Age	−.214	.087
Gender, male	.352	.130
Education	.407	.274
FT3	.318	.572
FT4	.389	.415
TSH	.436	.383
FBG	.311	.256
HbA1c	.394	.428
LDL‐C	.323	.149
TG	.265	.601
TC	.450	.513
HDL‐C	−.276	.395
NPTX2	.347	.042

Abbreviations: FBG, fasting blood glucose; FT3, free triiodothyronine 3; FT4, free triiodothyronine 4; HbA1c, hemoglobin A1c; HDL‐C, high‐density lipoprotein cholesterol; LDL‐C, low‐density lipoprotein cholesterol; MoCA, Montreal cognitive assessment; NPTX2, neuronal pentraxin 2; TC, total cholesterol; TG, triglycerides; TSH, thyroid‐stimulating hormone; VaD, vascular dementia.

### 3.3 Multivariate regression analysis

Multivariate regression analysis was used to evaluate the effect of clinical baseline data on VaD patients' MoCA score (Table 3). The results showed that serum NPTX2 level was an independent risk factor for VaD patients' cognitive function, even after adjusting for clinical baseline data including age, gender, education, FT3, FT4, TSH, FBG, HbA1c, LDL‐C, TC, TG, and HDL‐C, this predictive value still exists (*β* = 0.346, *p* = .039).

**TABLE 3:** Multivariable analysis between MoCA and baseline characteristics in patients with VaD

	Regression coefficient	*p*	95% CI
Age	0.363	.119	0.736–1.062
Gender, male	0.284	.162	0.583–1.197
Education	0.327	.254	0.865–1.058
FT3	0.451	.387	0.914–1.206
FT4	0.382	.435	0.832–1.145
TSH	0.238	.206	0.879–1.156
FBG	0.174	.293	0.631–1.039
HbA1c	0.229	.158	0.710–1.104
LDL‐C	0.315	.427	0.826–1.217
TG	0.341	.370	0.739–1.060
TC	0.143	.608	0.927–1.149
HDL‐C	0.212	.275	0.883–1.241
NPTX2	0.346	.039	0.562–0.913

Abbreviations: CI, confidence interval; FBG, fasting blood glucose; FT3, free triiodothyronine 3; FT4, free triiodothyronine 4; HbA1c, hemoglobin A1c; HDL‐C, high‐density lipoprotein cholesterol; LDL‐C, low‐density lipoprotein cholesterol; MoCA, Montreal cognitive assessment; NPTX2, neuronal pentraxin 2; TC, total cholesterol; TG, triglycerides; TSH, thyroid‐stimulating hormone; VaD, vascular dementia.

## 4 DISCUSSION

The main finding of this study is that VaD patients have lower serum NPTX2 levels than normal controls, and this serum NPTX2 levels are also positively correlated with VaD patients' MoCA scores. The association is independent of the effects of age, gender, education, FT3, FT4, TSH, FBG, HbA1c, LDL‐C, TC, TG, and HDL‐C. As far as I know, there has been no research report on the correlation between serum NPTX2 level and the cognitive function of VaD patients. The findings of this study can be extended to patients with the same characteristics.

The relationship between NPTX2 and a number of neurological diseases has been widely reported. One study had shown that the levels of NPTX2 in the inferior colliculus of audiogenic seizures (AGS)‐susceptible P77PMC rats were significantly increased after AGS, suggesting that NPTX2 is involved in the pathogenesis of AGS (Li, Xu, & Jia, 2003). Not only in epilepsy, Lang and his colleagues found that NPTX2 plays an important role in Parkinson's disease. Their results show that HOX transcript antisense intergenic RNA (HOTAIR) can increase the expression of NPTX2 in the substantia nigra through microRNA‐221–3, thereby triggering excessive autophagy of dopamine neurons (Lang et al., 2020). This further enriches the mechanisms by which NPTX2 participates in the pathogenesis of Parkinson's disease. Interestingly, studies have also shown that high levels of NPTX2 expression may be a biomarker for poor prognosis in human neuroblastoma (Bartolini et al., 2015). However, our results indicate that serum NPTX2 is decreased in VaD, which expands the spectrum of neurological diseases that NPTX2 may affect.

In recent years, NPTX2 has been confirmed to be closely related to the pathogenesis of AD. Galasko and his colleagues found that the levels of cerebrospinal fluid (CSF) synaptic protein NPTX2 in AD patients significantly decreased, and NPTX2 can be used as a biomarker for the progression of cognitive and global decline (Galasko et al., 2019). Further research shows that the predictive effect of NPTX2 is superior to the traditional biomarkers Aβ_1‐42_ and Tau. Iowa State University's research also shows that high baseline levels of NPTX2 in AD may have neuroprotective effects and can predict lower degrees of medial temporal lobe atrophy and cognitive decline (Swanson & Willette, 2016). Neuropathologists found that the expression of NPTX2 decreased significantly in the cerebral cortex of autopsy in AD patients (Hendrickson et al., 2015). All the above studies show that low levels of NPTX2 are involved in the pathogenesis of AD.

In addition to AD, NPTX2 is also associated with other forms of cognitive disorders. A newly published study points out that NPTX2 is significantly lower in genetic frontotemporal dementia (FTD) and is a novel synaptic marker to predict the progression of FTD disease (van der Ende et al., 2020). The results of BIOCARD Research Team show that the expression level of NPTX2 in mild cognitive impairment (MCI) is significantly lower than that of normal people, and NPTX2 may participate in the regulation of cognitive tasks in MCI (Soldan et al., 2019). Similar to the above studies, we found that serum NPTX2 levels of VaD patients were significantly lower than those of normal controls. Therefore, decreased NPTX2 may be associated with a variety of cognitive disorders.

Although some studies have observed fluctuations in the level of NPTX2 in various neurological diseases, the role of NPTX2 in it is largely unknown. NPTX2 can enhance the expression of brain‐derived neurotrophic factor (BDNF), while BDNF with neuroprotective effect can also promote the expression of NPTX2 (Mariga et al., 2015). The protective effect of NPTX2 on cognition depends on the synapses (Gu et al., 2013; Miskimon et al., 2014). Under normal circumstances, NPTX2 is secreted into the synapse and cannot adhere to the cell surface. The perineuronal net (PNN), a proteoglycan network structure on cell surface, can capture NPTX2 to the presynaptic membrane and postsynaptic membrane to regulate synaptic homeostasis and plasticity (Van't Spijker et al., 2019). However, the specific mechanism by which NPTX2 regulates synapses has not been fully elucidated.

There are several limitations in our study. First of all, we did not record the duration of VaD, so it is impossible to clarify the effect of different duration of VaD on the expression of NPTX2. Second, we did not dynamically observe the NPTX2 expression levels and MoCA scores of all the subjects. Third, there may be a mixed type of dementia among VaD subjects. Finally, it is a single‐center clinical study, and the findings may not apply to people in other regions or ethnic groups.

## 5 CONCLUSIONS

The conclusion of this study is that VaD patients have lower serum NPTX2 levels than normal controls, and serum NPTX2 levels are positively correlated with cognitive function. Moreover, the correlation between serum NPTX2 and cognitive function in VaD patients is independent of age, gender, education, FT3, FT4, TSH, FBG, HbA1c, LDL‐C, TC, TG, and HDL‐C. Therefore, NPTX2 may be a novel serum biomarker of VaD. It is expected that future research will further reveal the pathogenesis of NPTX2 in VaD, which will have important clinical significance.

## 6 Funding statement

This research was not funded.

## CONFLICT OF INTERESTS

The authors declare no conflict of interests.

## AUTHOR CONTRIBUTION

Keke Shao and Cuihua Ma designed the experiments. Keke Shao, Shiqin Shan, and Wenwen Ru participated in the cognitive assessment and ELISA experiments. Keke Shao drafted the manuscript and analyzed the data. Cuihua Ma edited the manuscript.

## Data Availability

The data used to support the findings of this study are available from the corresponding author upon request.

## References

[brb31779-bib-0001] Bartolini, A. , Di Paolo, D. , Noghero, A. , Murgia, D. , Sementa, A. R. , Cilli, M. , … Marchio, S. (2015). The neuronal pentraxin‐2 pathway is an unrecognized target in human neuroblastoma, which also offers prognostic value in patients. Cancer Research, 75(20), 4265–4271. 10.1158/0008-5472.can-15-0649 26294210

[brb31779-bib-0002] Chapman, G. , Shanmugalingam, U. , & Smith, P. D. (2019). The role of neuronal pentraxin 2 (NP2) in regulating glutamatergic signaling and neuropathology. Front Cell Neurosci, 13, 575 10.3389/fncel.2019.00575 31969807 PMC6960182

[brb31779-bib-0003] Galasko, D. , Xiao, M. , Xu, D. , Smirnov, D. , Salmon, D. P. , Dewit, N. , … Worley, P. (2019). Synaptic biomarkers in CSF aid in diagnosis, correlate with cognition and predict progression in MCI and Alzheimer's disease. Alzheimers Dement (N Y), 5, 871–882. 10.1016/j.trci.2019.11.002 31853477 PMC6911971

[brb31779-bib-0004] Gu, Y. , Huang, S. , Chang, M. C. , Worley, P. , Kirkwood, A. , & Quinlan, E. M. (2013). Obligatory role for the immediate early gene NARP in critical period plasticity. Neuron, 79(2), 335–346. 10.1016/j.neuron.2013.05.016 23889936 PMC3804028

[brb31779-bib-0005] Hendrickson, R. C. , Lee, A. Y. H. , Song, Q. , Liaw, A. , Wiener, M. , Paweletz, C. P. , … Yates, N. A. (2015). High resolution discovery proteomics reveals candidate disease progression markers of alzheimer's disease in human cerebrospinal fluid. PLoS One, 10(8), e0135365 10.1371/journal.pone.0135365 26270474 PMC4535975

[brb31779-bib-0006] Hsu, Y. C. , & Perin, M. S. (1995). Human neuronal pentraxin II (NPTX2): Conservation, genomic structure, and chromosomal localization. Genomics, 28(2), 220–227. 10.1006/geno.1995.1134 8530029

[brb31779-bib-0007] Jia, J. , Wei, C. , Chen, S. , Li, F. , Tang, Y. I. , Qin, W. , … Gauthier, S. (2018). Efficacy and safety of the compound Chinese medicine SaiLuoTong in vascular dementia: A randomized clinical trial. Alzheimers Dement (N Y), 4, 108–117. 10.1016/j.trci.2018.02.004 29955654 PMC6021260

[brb31779-bib-0008] Kimoto, S. , Zaki, M. M. , Bazmi, H. H. , & Lewis, D. A. (2015). Altered markers of cortical γ‐aminobutyric acid neuronal activity in schizophrenia: Role of the NARP gene. JAMA Psychiatry, 72(8), 747–756. 10.1001/jamapsychiatry.2015.0533 26038830 PMC4734385

[brb31779-bib-0009] Lang, Y. , Li, Y. , Yu, H. , Lin, L. , Chen, X. , Wang, S. , & Zhang, H. (2020). HOTAIR drives autophagy in midbrain dopaminergic neurons in the substantia nigra compacta in a mouse model of Parkinson's disease by elevating NPTX2 via miR‐221‐3p binding. Aging (Albany NY), 12(9), 7660–7678. 10.18632/aging.103028 32396526 PMC7244061

[brb31779-bib-0010] Lee, S.‐J. , Wei, M. , Zhang, C. , Maxeiner, S. , Pak, C. H. , Calado Botelho, S. , … Südhof, T. C. (2017). Presynaptic neuronal pentraxin receptor organizes excitatory and inhibitory synapses. Journal of Neuroscience, 37(5), 1062–1080. 10.1523/jneurosci.2768-16.2016 27986928 PMC5296791

[brb31779-bib-0011] Li, S. Y. , Xu, D. S. , & Jia, H. T. (2003). AGS‐induced expression of Narp is concomitant with expression of AMPA receptor subunits GluR1 and GluR2 in hippocampus but not inferior colliculus of P77PMC rats. Neurobiology of Diseases, 14(3), 328–335. 10.1016/j.nbd.2003.08.010 14678750

[brb31779-bib-0012] Llorens, F. , Hermann, P. , Villar‐Piqué, A. , Diaz‐Lucena, D. , Nägga, K. , Hansson, O. , … Zerr, I. (2020). Cerebrospinal fluid lipocalin 2 as a novel biomarker for the differential diagnosis of vascular dementia. Nature Communications, 11(1), 619 10.1038/s41467-020-14373-2 PMC6992814 32001681

[brb31779-bib-0013] Mariga, A. , Glaser, J. , Mathias, L. , Xu, D. , Xiao, M. , Worley, P. , … Chao, M. V. (2015). Definition of a bidirectional activity‐dependent pathway involving BDNF and Narp. Cell Reports, 13(9), 1747–1756. 10.1016/j.celrep.2015.10.064 26655895 PMC4681298

[brb31779-bib-0014] Miskimon, M. , Han, S. , Lee, J. J. , Ringkamp, M. , Wilson, M. A. , Petralia, R. S. , … Reti, I. M. (2014). Selective expression of Narp in primary nociceptive neurons: Role in microglia/macrophage activation following nerve injury. Journal of Neuroimmunology, 274(1–2), 86–95. 10.1016/j.jneuroim.2014.06.016 25005116 PMC4152392

[brb31779-bib-0015] Moreno‐Rodriguez, M. , Perez, S. E. , Nadeem, M. , Malek‐Ahmadi, M. , & Mufson, E. J. (2020). Frontal cortex chitinase and pentraxin neuroinflammatory alterations during the progression of Alzheimer's disease. Journal of Neuroinflammation, 17(1), 58 10.1186/s12974-020-1723-x 32066474 PMC7025403

[brb31779-bib-0016] Osera, C. , Pascale, A. , Amadio, M. , Venturini, L. , Govoni, S. , & Ricevuti, G. (2012). Pentraxins and Alzheimer's disease: At the interface between biomarkers and pharmacological targets. Ageing Research Reviews, 11(2), 189–198. 10.1016/j.arr.2011.12.004 22186030

[brb31779-bib-0017] Pribiag, H. , & Stellwagen, D. (2014). Neuroimmune regulation of homeostatic synaptic plasticity. Neuropharmacology, 78, 13–22. 10.1016/j.neuropharm.2013.06.008 23774138

[brb31779-bib-0018] Soldan, A. , Moghekar, A. , Walker, K. A. , Pettigrew, C. , Hou, X. , Lu, H. , … Worley, P. (2019). Resting‐state functional connectivity is associated with cerebrospinal fluid levels of the synaptic protein NPTX2 in non‐demented older adults. Frontiers in Aging Neuroscience, 11, 132 10.3389/fnagi.2019.00132 31231205 PMC6568192

[brb31779-bib-0019] Swanson, A. , & Willette, A. A. (2016). Neuronal Pentraxin 2 predicts medial temporal atrophy and memory decline across the Alzheimer's disease spectrum. Brain, Behavior, and Immunity, 58, 201–208. 10.1016/j.bbi.2016.07.148 PMC5349324 27444967

[brb31779-bib-0020] Tang, C. Z. , Zhang, D. F. , Yang, J. T. , Liu, Q. H. , Wang, Y. R. , & Wang, W. S. (2019). Overexpression of microRNA‐301b accelerates hippocampal microglia activation and cognitive impairment in mice with depressive‐like behavior through the NF‐κB signaling pathway. Cell Death & Disease, 10(4), 316 10.1038/s41419-019-1522-4 30962417 PMC6453902

[brb31779-bib-0021] van der Ende, E. L. , Xiao, M. , Xu, D. , Poos, J. M. , Panman, J. L. , Jiskoot, L. C. , … van Swieten, J. C. (2020). Neuronal pentraxin 2: A synapse‐derived CSF biomarker in genetic frontotemporal dementia. Journal of Neurology, Neurosurgery and Psychiatry, 91(6), 612–621. 10.1136/jnnp-2019-322493 PMC7279197 32273328

[brb31779-bib-0022] Van't Spijker, H. M. , Rowlands, D. , Rossier, J. , Haenzi, B. , Fawcett, J. W. , & Kwok, J. C. F. (2019). Neuronal pentraxin 2 binds PNNs and enhances PNN formation. Neural Plasticity, 2019, 6804575 10.1155/2019/6804575 31772567 PMC6854953

[brb31779-bib-0023] Wang, Q. , Xu, Y. , Qi, C. , Liu, A. , & Zhao, Y. (2020). Association study of serum soluble TREM2 with vascular dementia in Chinese Han population. International Journal of Neuroscience, 130(7), 708–712. 10.1080/00207454.2019.1702548 31847649

[brb31779-bib-0024] Wang, Q. , Yang, W. , Zhang, J. , Zhao, Y. , & Xu, Y. (2020). TREM2 overexpression attenuates cognitive deficits in experimental models of vascular dementia. Neural Plasticity, 2020, 8834275 10.1155/2020/8834275 32617097 PMC7306072

[brb31779-bib-0025] Xu, Y. , Wang, Q. , Cui, R. , Lu, K. , Liu, Y. , & Zhao, Y. (2017). Uric acid is associated with vascular dementia in Chinese population. Brain Behav, 7(2), e00617 10.1002/brb3.617 28239527 PMC5318370

[brb31779-bib-0026] Xu, Y. , Wang, Q. , Qu, Z. , Yang, J. , Zhang, X. , & Zhao, Y. (2019). Protective effect of hyperbaric oxygen therapy on cognitive function in patients with vascular dementia. Cell Transplantation, 28(8), 1071–1075. 10.1177/0963689719853540 31134827 PMC6728711

[brb31779-bib-0027] Zhang, J. , Tang, L. , Hu, J. , Wang, Y. , & Xu, Y. (2020). Uric acid is associated with cognitive impairment in the elderly patients receiving maintenance hemodialysis‐A two‐center study. Brain Behav, 10(3), e01542 10.1002/brb3.1542 31985179 PMC7066348

